# Crystal structure of yeast monothiol glutaredoxin Grx6 in complex with a glutathione-coordinated [2Fe–2S] cluster

**DOI:** 10.1107/S2053230X16013418

**Published:** 2016-09-22

**Authors:** Mohnad Abdalla, Ya-Nan Dai, Chang-Biao Chi, Wang Cheng, Dong-Dong Cao, Kang Zhou, Wafa Ali, Yuxing Chen, Cong-Zhao Zhou

**Affiliations:** a Hefei National Laboratory for Physical Sciences at the Microscale and School of Life Sciences, University of Science and Technology of China, Hefei, Anhui 230027, People’s Republic of China

**Keywords:** glutaredoxin, iron–sulfur cluster, *Saccharomyces cerevisiae*, crystal structure, oxidoreductase

## Abstract

Glutaredoxins (Grxs) constitute a superfamily of proteins that perform diverse biological functions. The *Saccharomyces cerevisiae* glutaredoxin Grx6 not only serves as a glutathione (GSH)-dependent oxidoreductase and as a GSH transferase, but also as an essential [2Fe–2S]-binding protein. Here, the dimeric structure of the C-terminal domain of Grx6 (holo Grx6C), bridged by one [2Fe–2S] cluster coordinated by the active-site Cys136 and two external GSH molecules, is reported. Structural comparison combined with multiple-sequence alignment demonstrated that holo Grx6C is similar to the [2Fe–2S] cluster-incorporated dithiol Grxs, which share a highly conserved [2Fe–2S] cluster-binding pattern and dimeric conformation that is distinct from the previously identified [2Fe–2S] cluster-ligated monothiol Grxs.

## Abstract 2

The dimeric structure of the C-terminal domain of Grx6, bridged by one [2Fe–2S] cluster coordinated by the active-site Cys136 and two external glutathione molecules, is reported.

## 1. Introduction   

Glutaredoxins (Grxs) are thioredoxin-fold oxidoreductases that utilize glutathione (GSH) as the reductant to maintain redox homeostasis in all kingdoms of life (Li, 2014 ▸). According to their active-site sequences, Grxs were initially classified as classic dithiol Grxs with a C*XX*C motif and monothiol Grxs containing a C*XX*S motif. A subsequent thorough comparative genomic analysis identified six families of Grxs, among which classes I and II are the most widespread (Rouhier *et al.*, 2010 ▸). Class I proteins contain both dithiol (CP/G/S/FYC, 2-C-Grxs) and monothiol (CP/SYS, 1-C-Grxs) Grxs. Class II proteins are 1-C-Grxs that harbour a strict CGFS motif. Generally, 2-C-Grxs contribute greatly to reducing protein–GSH mixed disulfides and intermolecular or intramolecular disulfides, whereas most 1-C-Grxs show decreased activities in catalyzing thiol–disulfide exchange reactions but adopt more specialized functions in cells (Manta *et al.*, 2013 ▸). For instance, class II 1-C-Grxs can coordinate [2Fe–2S] iron–sulfur clusters (ISCs), evolutionarily ancient prosthetic groups that are necessary to maintain essential life processes (Zhang, 2015 ▸). Interestingly, several 2-C-Grxs such as human Grx2 (CSYC motif) and poplar GrxC1 (CGYC motif) have also been found to bind ISC (Comini *et al.*, 2013 ▸).

The yeast *Saccharomyces cerevisiae* encodes eight Grxs (Grx1–Grx8) with diverse subcellular localizations and functions. In detail, Grx1 and Grx2 are cytosolic 2-C-Grxs harbouring a CPYC motif that are responsible for resistance to various hydroperoxides (Collinson *et al.*, 2002 ▸). Grx3, Grx4 and Grx5 are all 1-C-Grxs containing a CGFS motif for ISC coordination. Grx3 and Grx4 take part in regulation of iron uptake in the nucleus by interacting with the transcriptional activator Aft1, whereas Grx5 is required for ISC biogenesis in mitochondria (Tamayo *et al.*, 2016 ▸). Grx6 and Grx7 belong to the class I 1-C-Grxs with a CSYS and a CPYS motif, respectively; they are integral components of ER/Golgi membranes and are associated with the early secretory pathway (Lee *et al.*, 2007 ▸). Grx8 is a cytosolic 2-C-Grx with a CPDC motif and has a much lower GSH-dependent oxido­reductase activity (Tang *et al.*, 2014 ▸).

We have been working on yeast Grxs to reveal their structural and biochemical characteristics (Li, 2014 ▸; Luo *et al.*, 2010 ▸; Tang *et al.*, 2014 ▸). To date, we have solved the crystal structures of yeast Grx1 (PDB entries 3c1r and 3c1s; Yu *et al.*, 2008 ▸), Grx2 (PDB entries 3ctf and 3ctg; Li *et al.*, 2010 ▸), Grx5 (PDB entry 3gx8; Y. Wang, Y.-X. He, J. Yu, Y. Xiong, Y. Chen & C.-Z. Zhou, unpublished work), the C-terminal domain of Grx6 (PDB entry 3l4n; Luo *et al.*, 2010 ▸) and Grx8 (PDB entry 2m80; Tang *et al.*, 2014 ▸). In contrast to the other Grxs, Grx6 consists of three segments: a putative signal peptide (Met1–Ile36), an N-terminal domain (Lys37–Thr110) and a C-terminal Grx domain (Lys111–Asn231, designated Grx6C). It is worth mentioning that Grx6, which is more similar in activity to 2-C-Grxs than to other 1-C-Grxs, has a lower glutathione disulfide reductase activity but a higher glutathione transferase activity compared with yeast Grx1 (Luo *et al.*, 2010 ▸). It has been suggested that Grx6 could modulate the glutathionylation state of target proteins at the ER/Golgi lumen and consequently regulate the glutathione redox balance (Puigpinós *et al.*, 2015 ▸). Moreover, Grx6 is capable of bridging ISCs through its C-terminal domain, which possesses a unique motif of a two-strand antiparallel β-sheet beyond the typical Grx fold. Although the *in vivo* function of Grx6 binding to ISC remains to be elucidated, it is strongly indicated that Grx6 could serve as a trifunctional Grx in the cell, defining it as the head of a novel Grx subfamily (Luo *et al.*, 2010 ▸).

However, the structural features of the coordination of the ISC in Grx6 are still unknown. Here, we report the dimeric crystal structure of a [2Fe–2S] cluster-bound form of Grx6C (designated holo Grx6C) at 2.46 Å resolution. Holo Grx6C comprises a subunit-bridging [2Fe–2S] cluster that is ligated by the catalytic Cys136 residues of each subunit and two external GSH molecules. It adopts a highly similar ISC-binding pattern and dimeric conformation to the holo structure of human Grx2 (PDB entry 2ht9; Johansson *et al.*, 2007 ▸), but differs from the monothiol human Grx5 (CGFS motif; PDB entry 2wul; Johansson *et al.*, 2011 ▸), which displays a more extended conformation. Structure-based multiple-sequence alignment implied that holo Grx6C is more similar to ISC-linked 2-C-Grxs compared with class II 1-C-Grxs. In summary, yeast Grx6 is probably not only an oxidoreductase, but is also an ISC storage or delivery protein in the cell.

## 2. Materials and methods   

### 2.1. Cloning, expression and purification of Grx6   

**Figure 1:**
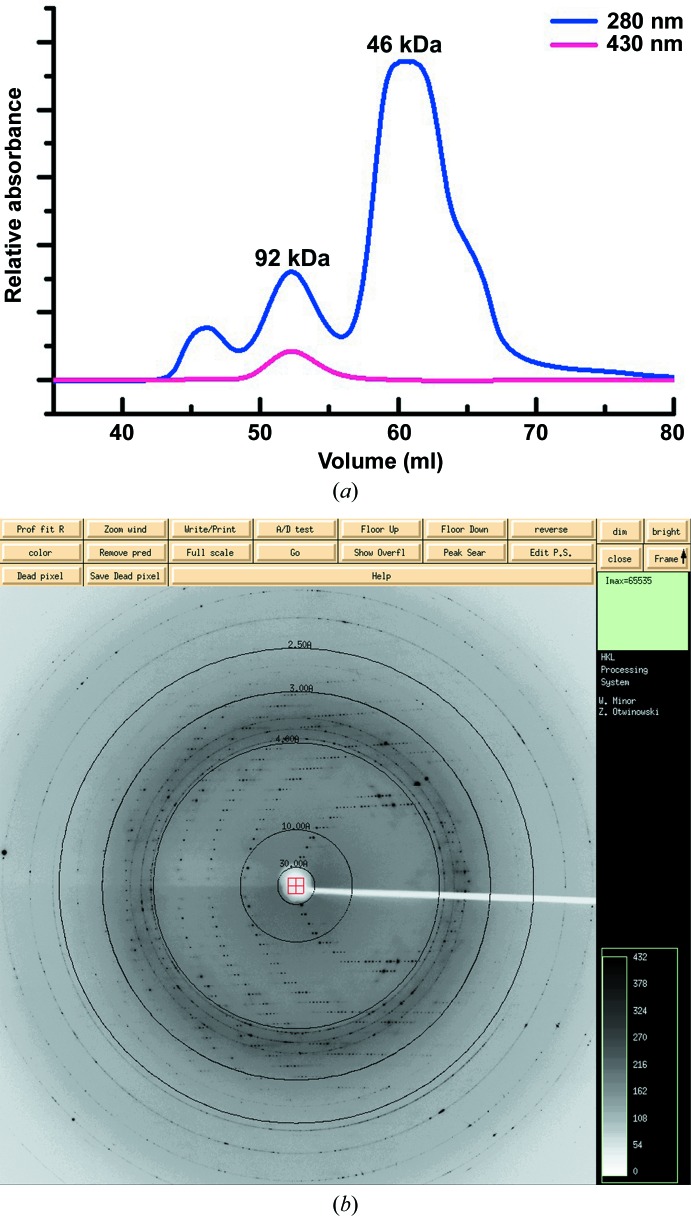
(*a*) Gel-filtration chromatography of full-length Grx6 and (*b*) a representative diffraction image for data collection from full-length Grx6.

The coding region of Grx6/YDL010W without the signal peptide (residues Lys37–Asn231, designated Grx6) was PCR-amplified from *S. cerevisiae* S288c genomic DNA and cloned into the pET-22b(+) vector (Novagen) with a C-terminal His_6_ tag. The recombinant plasmid was transformed into *Escherichia coli* strain BL21 (DE3) (Novagen) cultured in LB culture medium (10 g NaCl, 10 g Bacto tryptone and 5 g yeast extract per litre) containing 50 mg ml^−1^ ampicillin at 37°C. The expression of recombinant protein was induced using 0.02 m*M* IPTG at 16°C for 20 h after the cells had grown to an OD_600 nm_ of 0.8. Cells were collected by centrifugation and resuspended in buffer (20 m*M* NaCl, 50 m*M* Tris–HCl pH 8.0). After 20 min of sonication followed by centrifugation at 12 000*g* for 25 min, the supernatant was pooled and loaded onto an Ni–NTA column (GE Healthcare). The target protein was eluted with 250 m*M* imidazole and loaded onto a HiLoad 16/60 Superdex 75 column pre-equilibrated with buffer (20 m*M* NaCl, 50 m*M* Tris–HCl pH 8.0, 2 m*M* EDTA, 10 m*M* GSH). Similar to a previous report (Luo *et al.*, 2010 ▸), gel-filtration chromatography of Grx6 showed separated peaks representing distinctive oligomeric states (Fig. 1 ▸ *a*). The protein from the peak with a strong absorbance at 430 nm owing to the presence of the ISC was pooled and concentrated to 10 mg ml^−1^ for crystallization screening.

### 2.2. Crystallization, data collection and processing   

Grx6 crystals were grown at 14°C by the sitting-drop vapour-diffusion method using 1.0 µl protein sample, prepared as described above, and an equal volume of reservoir solution (0.1 *M* HEPES sodium pH 7.5, 0.8 *M* potassium sodium tartrate tetrahydrate). After about 12 h, diamond-shaped crystals appeared. A single crystal was transferred to cryoprotectant (reservoir solution supplemented with 20% glycerol) and flash-cooled with liquid nitrogen. X-ray diffraction data were collected at 100 K in a liquid-nitrogen stream on beamline 17U at the Shanghai Synchrotron Radiation Facility using an ADSC Q315r CCD detector (Area Detector Systems Corporation). The data sets were processed and scaled using *HKL*-2000 (Otwinowski & Minor, 1997 ▸).

### 2.3. Structure determination and refinement   

The structure of holo Grx6C was determined by molecular replacement with *MOLREP* in *CCP*4 (Winn *et al.*, 2011 ▸) using the structure of yeast GSH–Grx6C (PDB entry 3l4n; Luo *et al.*, 2010 ▸) as a search model. Refinement was performed with *REFMAC*5 (Murshudov *et al.*, 2011 ▸) and the model was rebuilt interactively using *Coot* (Emsley *et al.*, 2010 ▸). Water molecules and ligands were then placed into the electron-density map. The structure was finally refined to 2.46 Å resolution with an *R* factor of 19.6% and an *R*_free_ of 23.0%. An overall assessment of model quality was performed using *MolProbity* (Chen *et al.*, 2010 ▸) and *PROCHECK* (Laskowski *et al.*, 1993 ▸). Data-collection and refinement statistics are listed in Table 1 ▸. All structural figures were prepared with *PyMOL* (Schrödinger).

## 3. Results and discussion   

### 3.1. Overall structure of holo Grx6C   

**Figure 2:**
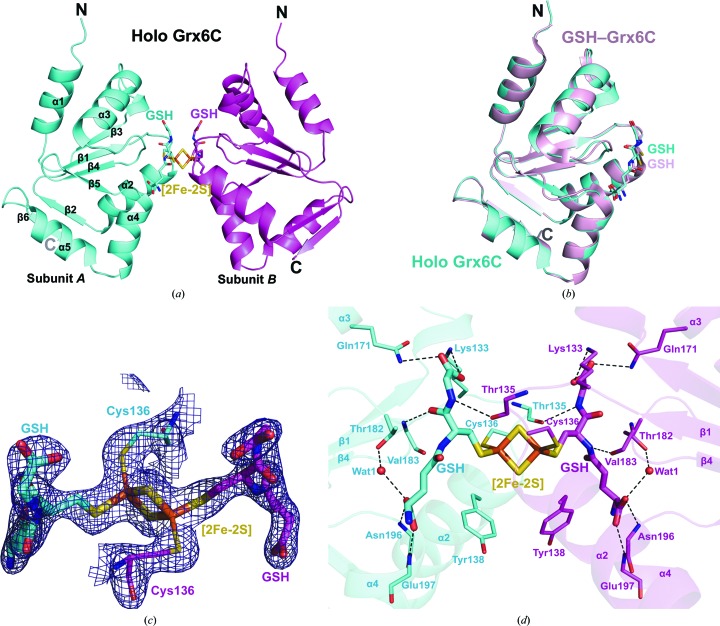
Crystal structure of yeast Grx6C bound to GSH and a [2Fe–2S] cluster. (*a*) The dimeric structure. Subunits *A* and *B* are coloured cyan and magenta, respectively. Secondary structures are labelled in subunit *A*. Two GSH molecules and the bridged [2Fe–2S] cluster are shown as sticks. S atoms are coloured yellow and Fe atoms brown. (*b*) Comparison of the overall structure between subunit *A* (cyan) of holo Grx6C and GSH–Grx6C (light magenta). (*c*) σ_A_-weighted 2*F*_o_ − *F*_c_ density map of Cys136 and the GSH-linked [2Fe–2S]. (*d*) Stereo representation of the [2Fe–2S] cluster coordination and GSH–Grx6C interactions. Two GSH molecules and the bridged [2Fe–2S] cluster are shown as bold sticks. Residues involved in GSH interaction are shown as sticks. Polar interactions are indicated by dashed lines.

Gel-filtration chromatography showed that full-length Grx6 eluted as three peaks (Fig. 1 ▸ *a*). The first peak corresponds to aggregated protein, and the other two correspond to apparent molecular masses of 46 kDa (dimer) and 92 kDa (tetramer). Protein from the peak with an absorbance at 430 nm, owing to the presence of the ISC, was used for crystallization. We obtained diamond-shaped crystals and they diffracted well. A representative diffraction image from the data collection is shown in Fig. 1 ▸(*b*). However, after structure solution only Grx6C could be built into the final model. An electrophoretic assay of the crystals confirmed that the protein had not been degraded during crystallization, indicating high flexibility of the N-terminal domain. Each asymmetric unit of the crystal contained one molecule of Grx6C. Analysis of the crystal packing suggests one [2Fe–2S] cluster-bridged homodimer with a buried surface area of 196 Å^2^ (Fig. 2 ▸ *a*). At a closer viewpoint on the dimeric interface, the [2Fe–2S] cluster is coordinated by the active-site Cys136 from each monomer in addition to two GSH molecules. Compared with the typical Grx fold, Grx6C comprises a two-stranded antiparallel β-sheet (β2 and β6) in addition to a central four-stranded antiparallel β-sheet (β1 and β3–β5) surrounded by five α-helices (α1–α5). The overall structures of holo Grx6C and GSH–Grx6C are quite similar, with a root-mean-square deviation of 0.28 Å over 100 C^α^ atoms (Fig. 2 ▸ *b*).

### 3.2. GSH/[2Fe–2S]-binding site   

The [2Fe–2S] cluster and two GSH models fit well in the electron-density map (Fig. 2 ▸ *c*). The pair of Fe atoms in the [2Fe–2S] cluster are coordinated by the S atoms of Cys136 from both subunits and two GSH molecules (Fig. 2 ▸ *c*). It is noted that the hydrophobic side chains of Tyr138 from both subunits contribute to the formation of the dimeric interface and help stabilize the cluster by reducing the solvent accessibility (Fig. 2 ▸ *d*).

GSH molecules are mainly fixed by a network of hydrogen bonds. Taking subunit *A* for instance, the GSH-binding groove mainly consists of the following parts: residues Asn196 and Glu197 from helix α4, Thr182 and Vla183 from the loop between α3 and β4, Thr135 from the loop connecting β1 and α2, and a patch composed of Lys133 and Gln171 (Fig. 2 ▸ *d*). In detail, Asn196 and Glu197 assist in the formation and orientation of helix α4, which is crucial for the proper positioning of GSH. The N^α^ atoms of both Asn196 and Glu197 form hydrogen bonds to the carboxyl group of the glutamyl moiety, which is further stabilized by the side chain of Thr182 *via* water-mediated (Wat1) hydrogen bonds. The cysteinyl carboxyl O atom is fixed by a hydrogen bond to the N^α^ atom of Val183. The N^α^ atom of the glycinyl moiety is stabilized by the main-chain O atom of Thr135 from the neighbouring subunit, whereas the carboxyl group of the glycinyl moiety is fixed by polar interactions with side chains of Lys133 and Gln171.

### 3.3. Comparison of Grx6 with ISC-ligated Grxs   

**Figure 3:**
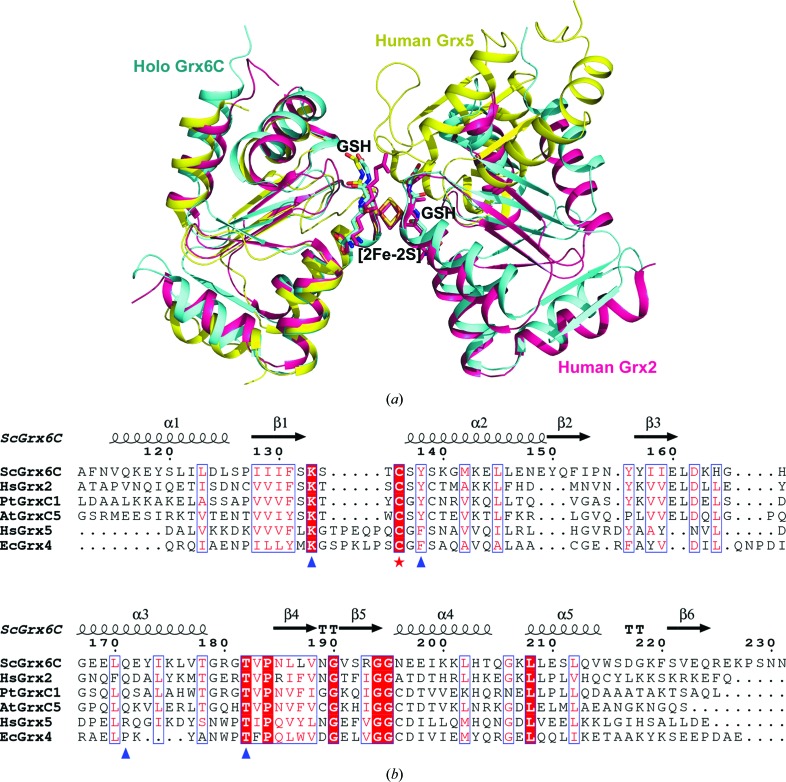
Comparison of structures of ISC-ligated Grxs. (*a*) Superposition of holo Grx6C (cyan) with human Grx2 (pink) and human Grx5 (yellow). (*b*) Multiple sequence alignment of solved structures of ISC-ligated Grxs. The active-site Cys136 and residues involved in GSH and [2Fe–2S] stabilization are indicated with a red star and blue triangles, respectively. The multiple sequence alignment was performed using *ESPript* (http://espript.ibcp.fr/ESPript/cgi-bin/ESPript.cgi). The sequences are *Saccharomyces cerevisiae* Grx6C (ScGrx6C), *Homo sapiens* Grx2 (HsGrx2), *Populus tremula* × *Populus tremuloides* GrxC1 (PtGrxC1), *Arabidopsis thaliana* GrxC5 (AtGrxC5), *Homo sapiens* Grx5 (HsGrx5) and *Escherichia coli* Grx4 (EcGrx4).

To date, five structures of ISC-ligated Grxs have been deposited in the PDB, including three class I 2-C-Grxs and two class II 1-C-Grxs. The 2-C-Grxs are human Grx2, poplar GrxC1 (PDB entry 2e7p; Rouhier *et al.*, 2007 ▸) and *A. thaliana* GrxC5 (PDB entry 3rhc; Couturier *et al.*, 2011 ▸), whereas the 1-C-Grxs include human Grx5 and *E. coli* Grx4 (PDB entry 2wci; Iwema *et al.*, 2009 ▸). Structural comparison demonstrated that holo Grx6C displays a dimeric conformation that is highly similar to that of human Grx2 but different from human Grx5 (Fig. 3 ▸ *a*), in which the conformation is more extended with more extensive intersubunit interactions. In addition, among the residues whose side chains stabilize GSH and the [2Fe–2S] cluster in holo Grx6C, Lys133 and Thr182 are highly conserved, whereas Tyr138 and Asn171 are more conserved in ISC-bound 2-C-Grxs than in 1-C-Grxs (Fig. 3 ▸ *b*). Taken together, Grx6 is more similar to ISC-linked 2-C-Grxs in both primary sequence and three-dimensional structure, compared with 1-C-Grxs harbouring the CGFS motif. The ISC has been suggested to function as a redox sensor for the activation of Grxs (Lillig *et al.*, 2005 ▸). For instance, human Grx2 becomes active upon loss of the [2Fe–2S] cluster *in vitro* by GSSG or *in vivo* by a decrease in the GSH:GSSG ratio (Lillig *et al.*, 2005 ▸). Therefore, Grx6 could also serve as an oxidoreductase regulated by the redox sensing of the incorporated ISC, in addition to the possibility of it being an ISC storage/delivery protein. Furthermore, the presence of a unique N-terminal domain indicates increased complexity of the cellular roles of Grx6, which calls for further functional explorations in the future. Nevertheless, the structural insights from holo Grx6C shed light on the diverse functions of yeast Grx6 to some degree.

## Supplementary Material

PDB reference: Grx6, complex with a glutathione-coordinated [2Fe–2S] cluster, 5j3r

## Figures and Tables

**Table 1:** Crystal parameters, data collection and structure refinement of holo Grx6C Values in parentheses are for the highest resolution bin.

Data collection	
Space group	*P*4_3_2_1_2
Unit-cell parameters (Å, °)	*a* = *b* = 58.657, *c* = 161.763, α = β = γ = 90.00
Resolution range (Å)	50.00–2.45 (2.54–2.45)
Wilson *B* factor (Å^2^)	45.0
Unique reflections	10929 (1055)
Completeness (%)	99.4 (99.5)
〈*I*/σ(*I*)〉	23.503 (8.455)
*R*_merge_ † (%)	8.1 (37.9)
Average multiplicity	12.6 (13.2)
Structure refinement	
Resolution range (Å)	28.96–2.46
*R* factor‡/*R*_free_ § (%)	19.6/23.0
No. of protein atoms	918
No. of ligands	2
No. of water molecules	53
R.m.s.d.¶, bond lengths (Å)	0.009
R.m.s.d., bond angles (°)	1.368
Mean *B* factors (Å^2^)	
Protein	45.1
Ligand	42.6
Water	50.5
Ramachandran plot	
Poor rotamers (%)	0
Most favoured (%)	99.11
Additionally allowed (%)	0.89
Outliers (%)	0
PDB entry	5j3r

† *R*_merge_ = 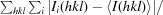 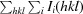, where *I_i_*(*hkl*) is the intensity of an observation and 〈*I*(*hkl*)〉 is the mean value for the unique reflection; summations are over all reflections.

‡ *R* factor = 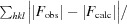 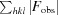, where *F*_obs_ and *F*_calc_ are the observed and calculated structure-factor amplitudes, respectively.

§ *R*_free_ was calculated with 5% of the data, which were excluded from the refinement.

¶ Root-mean-square deviation from ideal values.
